# Research on Nonlinear Error Compensation and Intelligent Optimization Method for UAV Target Positioning

**DOI:** 10.3390/s25144340

**Published:** 2025-07-11

**Authors:** Yinglei Li, Qingping Hu, Shiyan Sun, Wenjian Ying, Xiaojia Yan

**Affiliations:** Graduate School, Naval University of Engineering, 717 Jiefang Road, Qiaokou District, Wuhan 430030, China; lylxueshu@163.com (Y.L.); 0909061028@nue.edu.cn (S.S.); 18186644519@163.com (W.Y.); d23182604@nue.edu.cn (X.Y.)

**Keywords:** target localization, error allocation, Monte Carlo simulation, airborne optoelectronic pods

## Abstract

The realization of high-precision target positioning requires the systematic suppression of nonlinear perturbations in the UAV optoelectronic system and the optimization of the cumulative deviation of coordinate transformations through error transfer modeling. This study proposes an error allocation method based on the improved raccoon optimization algorithm (KYCOA) to resolve the problem of degradation of positioning accuracy due to multi-source error coupling during UAV target positioning. Firstly, a multi-coordinate system transformation model is established to analyze the nonlinear transfer characteristics of the error, and the Taylor expansion is used to linearize the error transfer process and derive the synthetic error model under the geocentric coordinate system. Secondly, the KYCOA is proposed to optimize the error allocation by combining the good point set initialization strategy to enhance the population diversity, and the golden sine algorithm to improve the position updating mechanism in response to the defect of the traditional optimization algorithm, which easily falls into the local optimum. Simulation experiments show that the positioning error distance of the KYCOA is reduced by 66.75%, 41.89%, and 62.06% when compared with that of the original Coati Optimization Algorithm (COA), Grey Wolf Optimizer (GWO), and Whale Optimization Algorithm (WOA), respectively. In the real flight test, the target point localization error of the KYCOA is reduced by more than 40% on average when compared with that of other algorithms, which verifies the effectiveness of the proposed method in improving the target localization accuracy and robustness of UAVs.

## 1. Introduction

With the wide application of unmanned technology in reconnaissance, monitoring, rescue, and other fields, its target localization accuracy has become a core performance indicator [1]. UAV target localization is divided into active localization and passive localization [2], but all types of localization methods have inherent defects, mainly associated with the accumulation of errors and algorithmic limitations [3]. Based on the needs of mission scale, UAVs can be divided into clustered collaborative systems [4,5] and single autonomous systems [6,7], and different localization algorithms and a number of UAVs aim to improve the estimation accuracy of the target position in the localization scenario.

A large number of researchers have carried out in-depth research on improving the accuracy of target localization results. Wang et al. [8] proposed a UAV target localization method based on depth estimation, which achieves higher accuracy in depth estimation. Zhou et al. [9] proposed a UAV cluster passive localization method based on the pheromone mechanism of an ant colony, which improves the target localization accuracy. Du et al. [10] proposed a direct-to-ground target localization algorithm for a time-delayed integral charge-coupled device panoramic aerial camera not equipped with a laser rangefinder. Wei et al. [11] proposed a dynamic UAV clustering model integrating a mobile sensing set, which is advantageous in terms of localization accuracy, computational efficiency, and adaptability to different UAV target scales. However, in the UAV target localization system, the localization accuracy is limited by the composite error produced by the coupling of multiple physical fields [12]. By applying Taylor series expansion to process these composite errors and utilizing an error propagation model to transform the equations into first-order Taylor series expansions, we can analyze the errors through coordinate system transformations using input parameters. This approach holds significant practical implications for enhancing the precision of UAV target positioning. Ren et al. [13] analyzed the sources of error in target positioning calculations by utilizing drone-related parameters and conducted an error analysis of target positioning. YARIMA et al. [14] conducted an error analysis of the target position estimation process using a multi-angle position estimation system. The above articles analyze errors in the drone target positioning process but do not apply the results to actual flight.

By synthesizing the error of the UAV during the positioning process into the overall error and optimizing it as a variable, the error assignment problem can be transformed into a parametric optimization problem and solved with an optimization algorithm. In many cases, traditional methods have often failed to provide the best results [15]. In recent years, the rise in meta-heuristic algorithms, such as particle swarm optimization algorithm (PSO) [16], ant colony optimization algorithm (ACO) [17], dung beetle optimization algorithm (DBO) [18], and others, has provided a new way of thinking for solving the optimization problem; among these algorithms, coati optimization algorithm (COA) [19] is inspired by coati’s hunting behavior. Due to its strong evolutionary ability, fast search speed, and strong optimality finding ability, it is widely used in various practical scenarios. Deng et al. [20] propose a multimodal multi-objective coati optimization algorithm based on spectral clustering for use in multimodal multi-objective problems. Jia et al. [21] improved the COA and applied it to 3D UAV path planning. Based on this, we attempted to apply the COA to the optimization of error allocation in drones.

However, there is no algorithm that can solve all problems efficiently. Each algorithm has difficulty maintaining generalization performance, and its optimization advantages can only be realized in local scenarios that meet preset adaptability conditions [22]. Therefore, researchers continue to invent new algorithms to address specific optimization problems. Zhou et al. [23] proposed an adaptive surrogate model-based swarm safe-migration planning method; its computational efficiency is faster than traditional GA methods. Peng et al. [24] proposed an adaptive surrogate model that has advantages over the direct global optimization method. In this study, we introduce a set of good points to increase the initialization of the population; at the same time, we introduce the golden sinusoidal strategy to enhance its exploratory ability, and the simulations and experiments show that the improved COA improves the optimality-finding results of dealing with the corresponding problems.

The main innovations of this study are as follows:

To address the complexity of multi-source error nonlinear transfer in UAV localization, an analytical model of error transfer based on Taylor expansion is proposed.

In view of the defects of the traditional COA, the optimization mechanism of the raccoon optimization algorithm is improved by introducing the good point set and golden sine strategy to improve the optimization ability.

The dual-flow verification of simulation and experiment proves that the improved COA obtains better results while dealing with the UAV error optimization problem.

The main structure of this paper is as follows: Section 2 derives the coordinate system transformation and error model; Section 3 analyzes the error transfer process; Section 4 describes the COA and proposes improvement measures; Section 5 and Section 6 verify the effectiveness of the COA through simulation and experiments, and Section 7 summarizes the research conclusions.

## 2. Principles of UAV Target Localization

### 2.1. Base Coordinate System

**Figure 1:**
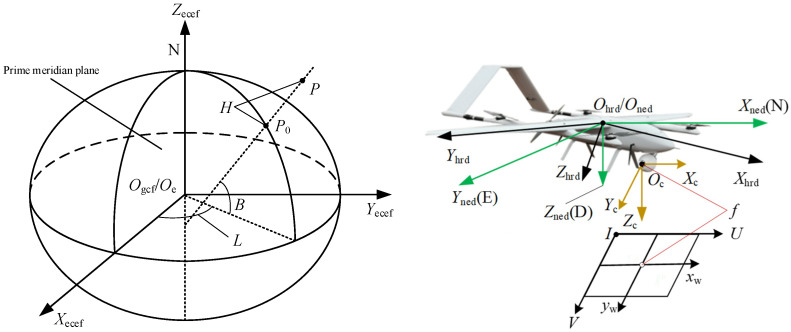
Basic coordinate system.

The basic coordinate systems that UAVs need to use in the target localization process are defined as follows: the geodetic coordinate system GCF $\left(O_{gcf}-BLH\right)$, the Earth-centered coordinate system ECEF $\left(O_{e}-X_{e}Y_{e}Z_{e}\right)$, the north-east-down coordinate system NED $\left(O_{ned}-X_{ned}Y_{ned}Z_{ned}\right)$, the airframe coordinate system HRD $\left(O_{hrd}-X_{hrd}Y_{hrd}Z_{hrd}\right)$, the camera coordinate system C $\left(O_{c}-X_{c}Y_{c}Z_{c}\right)$, and the image coordinate system I (I−UV), as shown in Figure 1.

### 2.2. Transformation of Coordinate System

The geodetic coordinate system of the UAV is known to be (B,L,H), the azimuth, pitch, and roll angles of the UAV are (φ,θ,σ), the azimuth is turned positive right up to the range of −180°∼180°, the pitch angle is turned positive up to the range of −180°∼180°, and the roll angle is turned positive right up to the range −180°∼180°. The on-board camera azimuth and pitch angle is (α,β); the azimuth right turn is positive, and the range is −180°∼180°; the pitch angle is positive, and the range is −180°∼0°. The image coordinates of the target point are (u,v), the physical size of a single image element of the camera in the direction of the x,y axes is dx,dy, and the focal length of the camera is f; then, the coordinate transformation process is shown below [25].

The coordinate transformation from the geodetic to the geocentric coordinate system is as follows:

(1) $$\left\{\begin{array}{c} X_{e}=(N+H)\cos(B)\cos(L)\\ Y_{e}=(N+H)\cos(B)\sin(L)\\ Z_{e}=[N(1-e^{2})+H]\sin(B) \end{array}\right.$$

where *N* denotes the radius of curvature in prime vertical, $N=a/\sqrt{1-e^{2}\sin^{2}(B)}$, and *a* and *e* denote the long radius and the first eccentricity of the Earth’s ellipsoid, respectively.

Assuming that $t_{c}=\left[\begin{array}{c} (u-u_{0})d_{x}\\ (v-v_{0})d_{y}\\ f \end{array}\right]$ is a vector in the on-board camera coordinate system and $t_{n}=\left[\begin{array}{c} 0\\ 0\\ 1 \end{array}\right]$ is a vector in the body coordinate system, then the representation of $t_{c}$ in the body coordinate system $t_{cn}$ is as follows [26]:

(2) $$t_{cn}=T_{f7}\cdot T_{f6}\cdot T_{f5}\cdot T_{f4}\cdot T_{f3}\cdot t_{c}$$

Then, the angle between the drone and the target point ϖ is as follows:

(3) $$\varpi =\arccos\frac{t_{cn}\cdot t_{n}}{\left|t_{cn}\right|\cdot \left|t_{n}\right|}$$

Therefore, the coordinates of the target point under the on-board camera coordinate system are shown in Equation (4):

(4) $$\left\{\begin{array}{l} \begin{array}{c} x_{c}=\frac{(u-u_{0})d_{x}\cdot H}{\cos\omega \cdot \sqrt{x_{w}^{2}+y_{w}^{2}+f^{2}}}\\ y_{c}=\frac{(v-v_{0})d_{y}\cdot H}{\cos\omega \cdot \sqrt{x_{w}^{2}+y_{w}^{2}+f^{2}}} \end{array}\\ z_{c}=\frac{f\cdot H}{\cos\omega \cdot \sqrt{x_{w}^{2}+y_{w}^{2}+f^{2}}} \end{array}\right.$$

where $\left(u_{0},v_{0}\right)$ represent the coordinates of the center point of the image.

To obtain the desired target point coordinates, it is necessary to perform a coordinate transformation from the on-board camera coordinate system to the geocentric coordinate system. The coordinate transformation matrix is shown below [27,28,29].

(5) $$T_{f1}=\left[\begin{array}{ccc} \cos(L) & \sin(L) & 0\\ -\sin(L) & \cos(L) & 0\\ 0 & 0 & 1 \end{array}\right]$$

(6) $$T_{f2}=\left[\begin{array}{ccc} -\sin(B) & 0 & \cos(B)\\ 0 & 1 & 0\\ -\cos(B) & 0 & -\sin(B) \end{array}\right]$$

(7) $$T_{f3}=\left[\begin{array}{ccc} \cos(\varphi ) & \sin(\varphi ) & 0\\ -\sin(\varphi ) & \cos(\varphi ) & 0\\ 0 & 0 & 1 \end{array}\right]$$

(8) $$T_{f4}=\left[\begin{array}{ccc} \cos(\theta ) & 0 & -\sin(\theta )\\ 0 & 1 & 0\\ \sin(\theta ) & 0 & \cos(\theta ) \end{array}\right]$$

(9) $$T_{f5}=\left[\begin{array}{ccc} 1 & 0 & 0\\ 0 & \cos(\sigma ) & \sin(\sigma )\\ 0 & -\sin(\sigma ) & \cos(\sigma ) \end{array}\right]$$

(10) $$T_{f6}=\left[\begin{array}{ccc} -\sin(\alpha ) & \cos(\alpha ) & 0\\ \cos(\alpha ) & \sin(\alpha ) & 0\\ 0 & 0 & 1 \end{array}\right]$$

(11) $$T_{f7}=\left[\begin{array}{ccc} 1 & 0 & 0\\ 0 & \cos(\beta ) & \sin(\beta )\\ 0 & -\sin(\beta ) & \cos(\beta ) \end{array}\right]$$

Based on Equations (2)–(11), the coordinate transformation from the on-board camera coordinate system to the geocentric coordinate system is obtained as follows:

(12) $$\left[\begin{array}{c} x_{e}\\ y_{e}\\ z_{e} \end{array}\right]={\left(T_{f2}\cdot T_{f1}\right)}^{-1}\left[{\left(T_{f5}\cdot T_{f4}\cdot T_{f3}\right)}^{-1}\left[{\left(T_{f7}\cdot T_{f6}\right)}^{-1}\left[\begin{array}{c} x_{c}\\ y_{c}\\ z_{c} \end{array}\right]+\left[\begin{array}{c} \Delta x\\ \Delta y\\ \Delta z \end{array}\right]\right]\right]+\left[\begin{array}{c} x_{e0}\\ y_{e0}\\ z_{e0} \end{array}\right]$$

where $\left[\begin{array}{c} \Delta x\\ \Delta y\\ \Delta z \end{array}\right]$ denotes the translation distance from the on-board camera to the center of the UAV, and $\left[\begin{array}{c} x_{e0}\\ y_{e0}\\ z_{e0} \end{array}\right]$ denotes the coordinates of the UAV in the geocentric coordinate system.

## 3. Error Analysis of Target Localization

In the process of drone target localization, the target position must be progressively transformed from the aircraft coordinate system to the geodetic coordinate system. This process involves the composite operation of rotation matrices and translation vectors, which inherently exhibit strong nonlinear characteristics. Due to the presence of sensor measurement errors, errors are coupled and amplified during nonlinear transmission, leading to the final localization results deviating from the true values. To suppress such error accumulation, a first-order linearization approximation of the nonlinear transfer function is performed using Taylor expansion, and the Jacobian matrix of error propagation is constructed to quantify the contribution of each error source to the composite positioning error in the geodetic coordinate system [30,31]. Based on this, a compensation algorithm is designed to correct the positioning results.

### 3.1. Error Transfer Process

The UAV is affected by a variety of errors in the target localization process, such as the UAV position error, UAV attitude angle error, and on-board camera azimuth and pitch angle error in the target localization process; all of the above errors are random errors, exhibiting a normal distribution and being potentially independent of each other.

If the probability density of random variable *X* is: $f(x)=\frac{1}{\sqrt{2\pi }\sigma }\exp(-\frac{{\left(x-\mu \right)}^{2}}{2\sigma^{2}})$, then *X* is said to follow a normal distribution, denoted as $X\sim N(\mu ,\sigma^{2})$.

Among them, −∞<μ<∞, σ>0, μ is the mean of the random variable *X*, and σ is the standard deviation of the random variable *X*.

Assuming that the target point *P* in the on-board camera coordinate system is $P_{c}=(x_{c},y_{c},z_{c})$, the camera azimuth angle is α, and the pitch angle β error is ${\left(\alpha ,\beta ,0\right)}^{T}∼N(\mu_{c},\sigma_{c})$, where $\mu_{c}={\left(\bar{\alpha },\bar{\beta },0\right)}^{T}$ and $\sigma_{c}=diag(\rho_{\alpha }^{2},\rho_{\beta }^{2},0)$, ρ is the standard deviation from the coordinate transformation that can be obtained from the target point *P* in the airframe coordinate system coordinates $P_{h}=(x_{h},y_{h},z_{h})$; then, the error transfer from the on-board camera coordinate system to the airframe coordinate system is expressed as follows:

(13) $$\Sigma_{h}=(J_{c}^{h})\sigma_{c}{\left(J_{c}^{h}\right)}^{T}$$

where

(14) $$J_{c}^{h}=\left[\begin{array}{ccc} \frac{\partial x_{h}}{\partial \alpha } & \frac{\partial x_{h}}{\partial \beta } & 0\\ \frac{\partial y_{h}}{\partial \alpha } & \frac{\partial y_{h}}{\partial \beta } & 0\\ \frac{\partial z_{h}}{\partial \alpha } & \frac{\partial z_{h}}{\partial \beta } & 0 \end{array}\right]$$

For the airframe coordinate system coordinates $P_{h}=(x_{h},y_{h},z_{h})$ and the UAV attitude angle ${\left(\phi ,\theta ,\sigma \right)}^{T}∼N(\mu_{h},\sigma_{h})$, where $\mu_{h}={\left(\bar{\phi },\bar{\theta },\bar{\sigma }\right)}^{T}$ and $\sigma_{h}=diag(\rho_{\phi }^{2},\rho_{\theta }^{2},\rho_{\sigma }^{2})$, the target point *P* in the navigation coordinate system coordinates $P_{n}=(x_{n},y_{n},z_{n})$ can be obtained from the coordinate transformation; then, the error transfer from the airframe coordinate system to the navigation coordinate system is expressed as follows:

(15) $$\Sigma_{n}=(J_{h}^{n})\left[\begin{array}{cc} \Sigma_{h} & 0\\ 0 & \sigma_{c} \end{array}\right]{\left(J_{h}^{n}\right)}^{T}$$

where

(16) $$J_{h}^{n}=\left[\begin{array}{ccc} \frac{\partial x_{n}}{\partial \phi } & \frac{\partial x_{n}}{\partial \theta } & \frac{\partial x_{n}}{\partial \sigma }\\ \frac{\partial y_{n}}{\partial \phi } & \frac{\partial y_{n}}{\partial \theta } & \frac{\partial y_{n}}{\partial \sigma }\\ \frac{\partial z_{n}}{\partial \phi } & \frac{\partial z_{n}}{\partial \theta } & \frac{\partial z_{n}}{\partial \sigma } \end{array}\right]$$

For the navigational coordinate system coordinates $P_{n}=(x_{n},y_{n},z_{n})$ and the UAV geodetic coordinates $U_{gfc}={\left(B,L,H\right)}^{T}∼N(\mu_{gfc},\sigma_{gfc})$, where $\mu_{gfc}={\left(\bar{B},\bar{L},\bar{H}\right)}^{T}$ and $\sigma_{gfc}=diag(\rho_{B}^{2},\rho_{L}^{2},\rho_{H}^{2})$, the geocentric coordinate system coordinates $P_{e}=(x_{e},y_{e},z_{e})$ can be obtained from the navigational coordinate system of the target point after the coordinate transformation.

Then, the error transfer from the navigation coordinate system to the geocentric coordinate system is expressed as follows:

(17) $$\Sigma_{e}=(J_{n}^{e})\left[\begin{array}{cc} \Sigma_{n} & 0\\ 0 & \sigma_{gfc} \end{array}\right]{\left(J_{n}^{e}\right)}^{T}$$

where

(18) $$J_{n}^{e}=\left[\begin{array}{ccc} \frac{\partial x_{e}}{\partial B} & \frac{\partial x_{e}}{\partial L} & \frac{\partial x_{e}}{\partial H}\\ \frac{\partial y_{e}}{\partial B} & \frac{\partial y_{e}}{\partial L} & \frac{\partial y_{e}}{\partial H}\\ \frac{\partial z_{e}}{\partial B} & \frac{\partial z_{e}}{\partial L} & \frac{\partial z_{e}}{\partial H} \end{array}\right]$$

### 3.2. Synthesis of Errors

According to Equations (13)–(18), the error matrix of the target coordinate position in the geocentric coordinate system after coordinate transformation is expressed as follows:

(19) $$\Sigma_{e}=J_{n}^{e}\left[\begin{array}{cc} J_{h}^{n}\left[\begin{array}{cc} J_{c}^{h}\sigma_{c}{\left(J_{c}^{h}\right)}^{T} & O\\ O & \sigma_{h} \end{array}\right]{\left(J_{h}^{n}\right)}^{T} & O\\ O & \sigma_{gfc} \end{array}\right]{\left(J_{n}^{e}\right)}^{T}$$

Therefore, the synthetic error of target localization in the geocentric coordinate system is expressed as follows:

(20) $$\sigma_{total}=\sqrt{trace(\Sigma_{e})}$$

where $trace(\Sigma_{e})$ represents the sum of the diagonal elements of the matrix $\Sigma_{e}$.

After completing the synthesis error calculation in the geocentric coordinate system, it is necessary to distinguish between the handling strategies for systematic errors and random errors. Systematic errors can be eliminated at the source through pre-calibration, so their influence can be ignored in the error allocation model; random errors, however, are unpredictable and will continuously affect the positioning process, leading to result deviations. To mitigate such errors, the error sources are modeled as optimizable variables, and their weights in the error propagation chain are adjusted through a parameter allocation mechanism. This problem can be transformed into a multi-parameter collaborative optimization problem, and intelligent optimization algorithms are introduced to solve the optimal allocation scheme, ultimately achieving dynamic compensation and improved accuracy of positioning results.

## 4. Assigning Errors Based on Improved Raccoon Optimization Algorithm

### 4.1. Target Allocation Model

The assessment of the positioning accuracy of the UAV can use the straight-line distance between the calculated point and the real position as an evaluation index. The magnitude of this distance value directly reflects the accuracy of the positioning result, and a shorter distance indicates that the estimated position matches the real position more closely. In the process of error allocation, it is also necessary to ensure that the error after allocation cannot exceed the total error of calculation [19]. In summary, the objective function and constraints are shown in Equation (21).

(21) $$\begin{array}{c} F=\min\{\sqrt{\left\|\vec{D}\right\|+{\left(H-H_{mean}\right)}^{2}}\}\\ s.t.\left\{\sigma_{toal}^{2}-\sigma_{xe}^{2}-\sigma_{ye}^{2}-\sigma_{ze}^{2}>0\right\} \end{array}$$

where $\left\|\vec{D}\right\|$ denotes the distance between the truth point and the latitude and longitude of the computation point, calculated using the spherical cosine theorem.

(22) $$\left\|\vec{D}\right\|=R*\arccos(\sin(B)\sin(B_{mean})+\cos(B)\cos(B_{mean})\cos(L-L_{mean}))$$

B and L denote the true latitude and longitude of the target point, $B_{mean}$ and $L_{mean}$ denote the average latitude and longitude of the computed point, $\sigma_{total}$ denotes the total error to be assigned, and $\sigma_{xe},\sigma_{ye},\sigma_{ze}$ denote the error assigned to the x,y,z axes.

### 4.2. Raccoon Optimization Algorithm

The coati optimization algorithm (COA) optimizes model parameters through two behaviors: cooperative iguana hunting and dispersal to escape from predators; the original coati optimization algorithm is briefly described as follows:

(1) Exploration phase—cooperative iguana hunting

After population initialization, half of the raccoons climb a tree to approach the iguana for hunting, while the other half of the raccoons gather under the tree and swim away to wait for the iguana to arrive; when the iguana arrive, the raccoons hunt it, and the iguana represents the global optimal position, and the tree-climbing raccoon expression is as follows:

(23) $$x_{i}^{t+1}=x_{i}^{t}(j)+r\cdot (x_{best}^{t}(j)-I\cdot x_{i}^{t}(j)),i=1,2,\cdots ,\frac{N}{2}$$

The iguana arrives at a random position, and the ground raccoon move randomly as a result, which is modeled mathematically as follows:

(24) $$x_{i}^{t+1}(j)=\left\{\begin{array}{l} \begin{array}{ll} x_{i}(j)+r\cdot (I_{gu}^{G}(j)-I\cdot x_{i}(j)), & \mathrm{if}\;\mathrm{fitness}(I_{gu}^{G})<\mathrm{fitness}(x_{i})\\ x_{i}(j)+r\cdot (x_{i}(j)-I_{gu}^{G}(j)), & \mathrm{otherwise} \end{array}\\ i=\frac{N}{2}+1,\frac{N}{2}+2,\cdots ,N \end{array}\right.$$

where *I* represents a randomly selected integer 1 or 2, and $I_{gu}^{G}(j)=lb_{j}+r\cdot (ub_{j}-lb_{j})$, r=0.5, denote scale factors.

(2) Development phase—dispersal to escape from predators

If there is a predator attacking the raccoon, the raccoon flees from its original location and seeks shelter in a nearby safe location, which is mathematically modeled as follows:

(25) $$x_{i}^{t+1}(j)=\left\{\begin{array}{l} x_{i}^{t}(j)+(1-2r)\left(lb_{j}^{\mathrm{local}}+r(ub_{j}^{\mathrm{local}}-lb_{j}^{\mathrm{local}})\right)\\ i=1,2,\cdots ,N\\ j=1,2,\cdots ,M \end{array}\right.$$

where $lb_{j}^{\mathrm{local}}=\frac{lb_{j}}{t},\;ub_{j}^{\mathrm{local}}=\frac{ub_{j}}{t},\;t=1,2,\cdots ,T$.

After each move, the position is updated using a greedy strategy as follows:

(26) $$x_{i}^{t+1}=\left\{\begin{array}{ll} x_{i}^{t+1}, & \mathrm{fitness}(x_{i}^{t+1})<\mathrm{fitness}(x_{i}^{t})\\ x_{i}^{t}, & \mathrm{fitness}(x_{i}^{t+1})\geq \mathrm{fitness}(x_{i}^{t}) \end{array}\right.$$

### 4.3. Improved Raccoon Optimization Algorithm

No single population intelligent optimization algorithm can solve all optimization problems; every population intelligent optimization algorithm has limitations and restrictions; in solving the problem of this study, the original raccoon algorithm encounters problems such as low convergence accuracy and prematurely falling into local optimal solutions; thus, it is decided to improve the raccoon optimization algorithm.

In the original raccoon optimization algorithm, the population initialization is in the form of random initialization; the randomly generated population is not uniformly distributed throughout the solution space, being very clustered in some regions and dispersed in others, resulting in the algorithm’s poor utilization of the entire search space and poor population diversity. In this section, the initialization of the population is optimized using the good point set. The Jia point set is a point set proposed by Hua Luogeng to satisfy the optimal distribution of point set coherence; its optimal distribution properties can make the population initialization more uniform, and its basic definition and properties, if there exists a point set, are as follows:

(27) $$P_{n}(k)=\left\{\left\{r_{1}^{\left(n\right)}\cdot k\right\},\left\{r_{2}^{\left(n\right)}\cdot k\right\},\cdots ,\left\{r_{s}^{\left(n\right)}\cdot k\right\}\right\},\;1\leq k\leq n$$

The deviation of this point set is satisfied as follows:

(28) $$\varphi (n)=C(r,\varepsilon )n^{-1+\varepsilon }$$

where C(r,ε) denotes a constant associated only with r and ε. Then, Equation (27) is called the set of good points, and a common way to construct the set of good points is shown in the following equation:

(29) $$r_{i}=2\cos\left(\frac{2\pi i}{p}\right),1\leq i\leq s$$

where *p* represents the smallest prime number that satisfies (p−3)/2≥s.

Therefore, based on the theory of good point sets, the new population initialization strategy is as follws:

(30) $$x_{i}(j)=(ub_{j}-lb_{j})\left\{r_{j}^{\left(i\right)}\cdot k\right\}+lb_{j}$$

**Figure 2:**
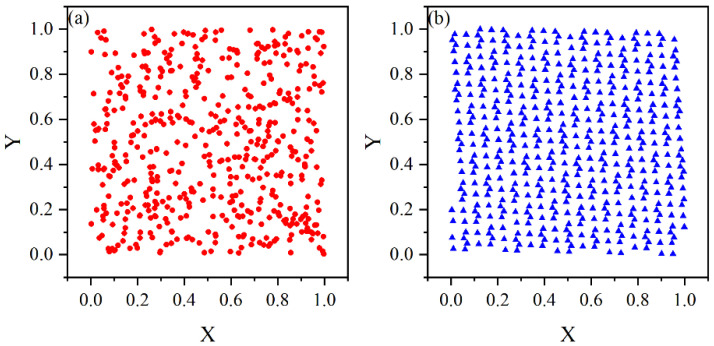
Comparison of population generation. (**a**) Randomized method. (**b**) Good point set method.

Subfigures (a) and (b) in Figure 2 show the schematic diagrams generated using the randomized method and the good point set method, respectively.

The golden sine algorithm is a novel meta-heuristic optimization algorithm proposed by Tanyildizi. The algorithm is based on the mathematical mapping relationship between the sine function and the unit circle and achieves the global traversal search of the solution space by establishing the correspondence between the search position and the point on the unit circle. The core mechanism utilizes the periodicity feature of the sine function, which enables the algorithm to systematically explore all potentially optimal regions in the solution space. This algorithm has two significant features: firstly, by introducing the golden section number as the coefficient parameter, the algorithm achieves the directional intensive search for the high-quality solution region during the position updating process, which significantly improves the development ability of the algorithm; secondly, based on the traversal characteristics of the unit circle, the algorithm has a good exploratory ability, which effectively avoids the phenomenon of premature convergence, which is expressed as the following specific form:

(31) $$X_{i}^{t+1}=X_{i}^{t}\cdot |\sin(r_{1})|+r_{2}\cdot \sin(r_{1})\cdot |x_{1}\cdot X_{best,i}^{t}-x_{2}\cdot X_{i}^{t}|$$

where $r_{1}$ denotes the random number from 0 to 2π, $r_{2}$ denotes the random number from 0 to π, $x_{1}=-\pi +(1-\tau )\cdot 2\pi$, $x_{2}=-\pi +\tau \cdot 2\pi$, $\tau =\frac{\sqrt{5}-1}{2}$ denotes the golden section ratio, and $X_{best}^{t}$ denotes the optimal value of the *tth* iteration.

The golden sine algorithm is introduced into the location update strategy for the exploration and development phases; thus, Equation (24) is updated to the following equation:

(32) $$x_{i}^{t+1}(j)=\left\{\begin{array}{l} \begin{array}{ll} x_{i}(j)\cdot |\sin(r_{1})|+r_{2}\cdot \sin(r_{1})\cdot |x_{1}\cdot I_{gu}^{G}(j)-x_{2}\cdot x_{i}(j)|, & \mathrm{if}\;\mathrm{fitness}(I_{gu}^{G})<\mathrm{fitness}(x_{i})\\ x_{i}(j)\cdot |\sin(r_{1})|+r_{2}\cdot \sin(r_{1})\cdot |x_{1}\cdot x_{i}(j)-x_{2}\cdot I_{gu}^{G}(j)|, & \mathrm{otherwise} \end{array}\\ i=\frac{N}{2}+1,\frac{N}{2}+2,\cdots ,N \end{array}\right.$$

Equation (25) is updated to the following equation:

(33) $$x_{i}^{t+1}(j)=\left\{\begin{array}{l} x_{i}^{t}\cdot |\sin(r_{1})|+r_{2}\cdot \sin(r_{1})\cdot |x_{1}\cdot X_{best,i}^{t}-x_{2}\cdot x_{i}^{t}|+(1-2r)\left(lb_{j}^{\mathrm{local}}+r(ub_{j}^{\mathrm{local}}-lb_{j}^{\mathrm{local}})\right)\\ i=1,2,\cdots ,N\\ j=1,2,\cdots ,M \end{array}\right.$$

**Figure 3:**
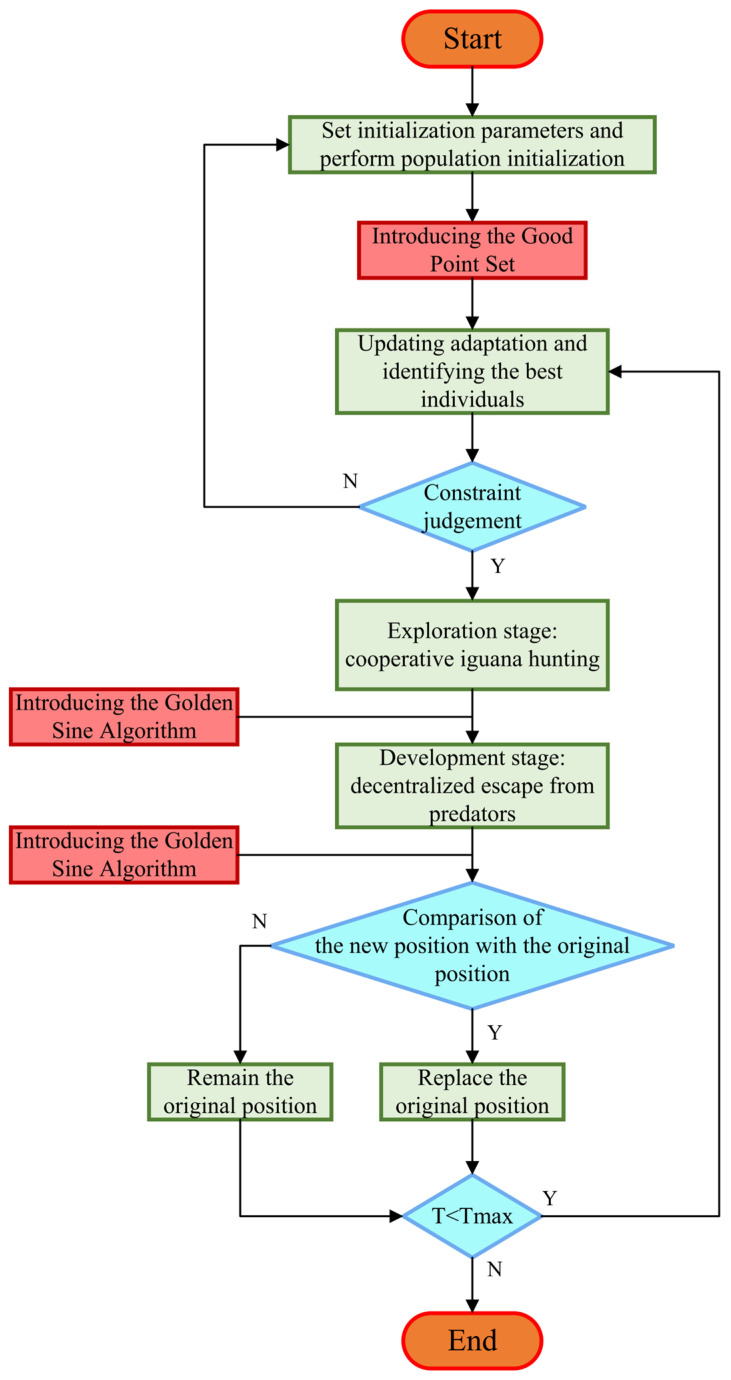
Flowchart of KYCOA.

The flowchart and pseudocode (Algorithm 1) of the KYCOA is shown in Figure 3.
**Algorithm 1.** KYCOA **Input**: *N* (coati size), *T* (number of iterations), *ub* (upper variable limits),
*lb* (lower variable limits), *dim* (variable dimensions)
 *F* = initialize(*N*) (Introduce good point set initialization)
 Updating adaptation and identifying the best individuals
 **While** *T* ≤ *T*max **do**
   Update location of the iguana based on best member location
   **Phase 1**
   **for** *i* = 1 **to** *N*/2 **do**
        Calculate new position for *i*-th coati using Equation (23)
   **end for**
   **for** *i* = *N*/2 + 1 **to** *N*
**do**
        Calculate random position for the iguana using Equation (24)
        Introducing the golden sine algorithm
        Update position of *i*-th coati using Equation (32)
   **end for**
   **Phase 2**
   **for** *i* = 1 **to** *N*
**do**
        Calculate new position for *i*-th coati using Equation (25)
        Introducing the golden sine algorithm
        Update position of *i*-th coati using Equation (33)
   **end for**
   Comparison of the new position with the original position
   *T* = *T* + 1
 **end while**
 **Output** the best solution
 **End**

## 5. Simulation Experiment

**Figure 4:**
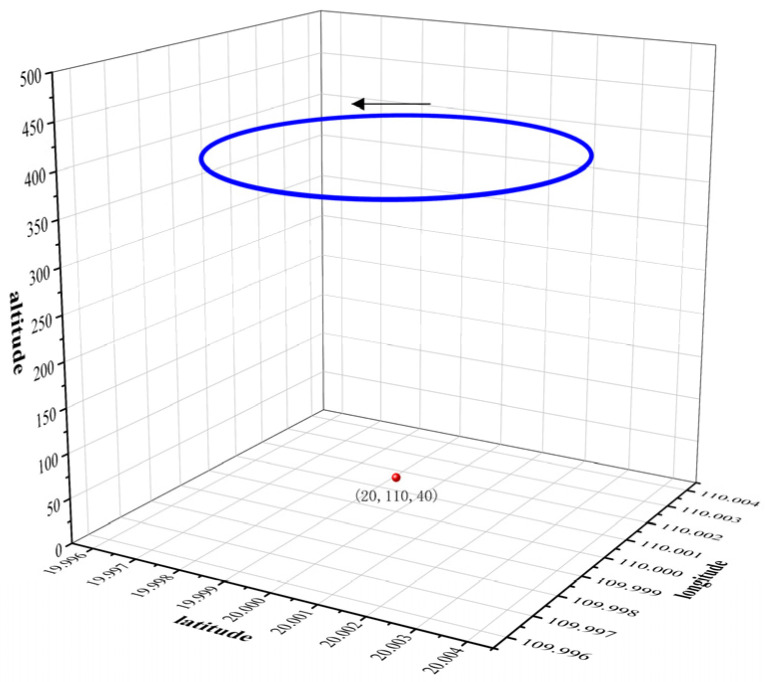
Drone simulation flight path diagram.

In order to verify the effect of the KYCOA proposed in this study on UAV target localization, the Monte Carlo simulation comparison experiments are performed. The UAV is centered at 20° N, 110° E, and it is moved counterclockwise in a circular motion at an altitude of 400 m with a circling radius of 400 m. The roll angle of the UAV is 20°, the pitch angle of the UAV is 0°, the heading angle of the pod is −90°, and the pitch angle of the pod is −25°. The simulated target point is set to be at an altitude of 40 m above sea level and is always located at the center of the camera’s field of view, and the flight path of the UAV is shown in Figure 4.

**Figure 5:**
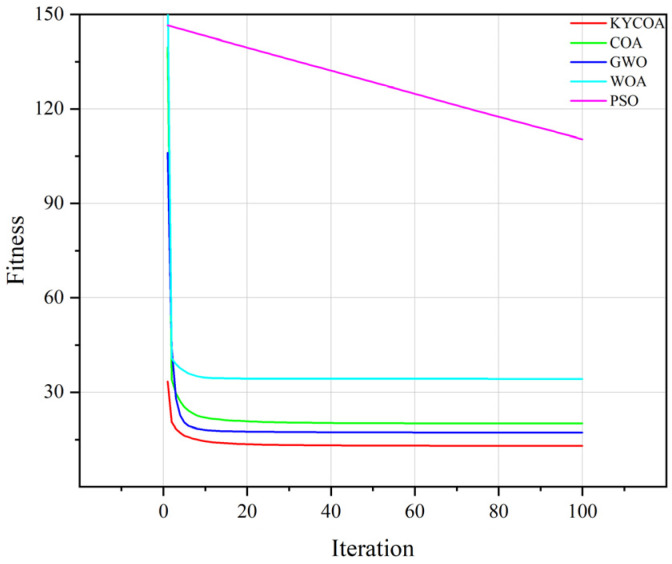
Simulation fitness curve.

The particle swarm optimization algorithm (PSO), Whale Optimization Algorithm (WOA), Gray Wolf Optimization Algorithm (GWO), and the original raccoon optimization algorithm (COA) are selected as the benchmark comparison algorithms for the experiment to assess the efficacy of the improved algorithms in terms of global optimization seeking ability and convergence accuracy. The various error simulation parameters of the UAV are shown in Table 1, and the experiment sets the population size to 30 and the number of iterations of the algorithm to 200; in order to verify the reliability of the simulation results, the average of the results of 100 simulation experiments is considered for drawing a change curve of fitness, as shown in Figure 5.

The results in Figure 5 show that the improved COA converges quickly when facing the problem and finally obtains the global optimal solution with the lowest fitness, which outperforms the other optimization algorithms, while the PSO algorithm does not converge in the specified number of iterations; therefore, it is not considered in the following experiments.

**Figure 6:**
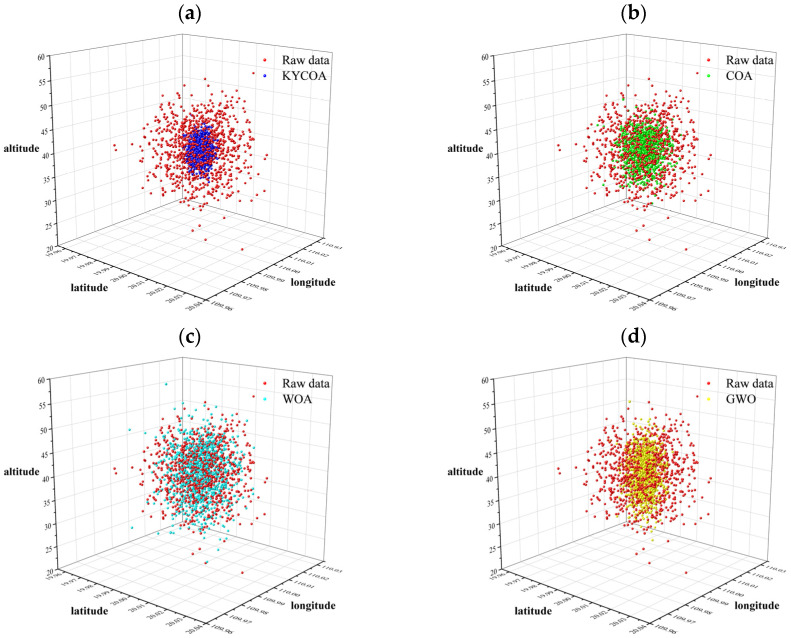
Scatter plot of target point localization results. (**a**) KYCOA-processed data. (**b**) COA-processed data. (**c**) WOA-processed data. (**d**) GWO-processed data.

The UAV is set to localize the target point 1000 times on the flight trajectory, and the UAV position, attitude angle, on-board camera azimuth, and pitch angle errors are generated by using the random number function provided by matlab and obeying the respective probability distributions. Figure 6 depicts the localization results of each algorithmic process in comparison with the original data, where (a) shows the KYCOA-processed data, (b) shows the COA-processed data, (c) shows the WOA-processed data, and (d) shows the GWO-processed data.

It is evident from Figure 6 that after the KYCOA processing, the target point positioning results are closer to the set true value. Then, the average of each algorithm is considered to locate the latitude, longitude, and altitude and compared with their true values, and the straight-line distance between the simulated average point and the true value is calculated using Formula (22), as shown in Table 2.

Table 2 visualizes that the KYCOA significantly improves the accuracy of UAV target localization by 66.75%, 41.89%, 62.06%, and 27.69%, respectively, compared to raw data, COA, WOA, GWO.

The performance of the algorithm is evaluated based on the root mean square error, which is defined as follows:

(34) $$RMSE=\sqrt{\frac{1}{n}\sum_{i=1}^{n}{\left(X-X_{i}\right)}^{2}}$$

where *n* represents the number of Monte Carlo simulations; X represents the real value of the target point; $X_{i}$ represents the simulated value of the target point. The root mean square error of the latitude, longitude, and altitude results of each algorithm is calculated, and the results are shown in Table 3.

It is evident from Table 3 that the proposed algorithm has the smallest root mean square error, which indicates that the proposed algorithm has high robustness and stability and proves that the KYCOA can effectively improve the results of target localization.

## 6. Actual Flight Test

**Figure 7:**
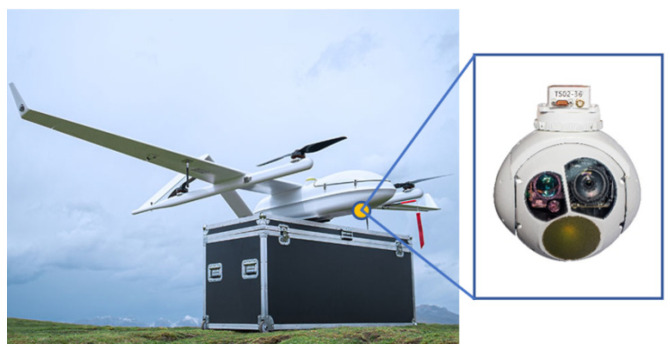
CW-25E UAV.

The experiment uses the CW-25E UAV produced by Chengdu Zongheng Automation Technology Co., Ltd. (Chengdu, China) and equipped with MG150E photoelectric pods (JOUAV, Chengdu City, China), which has the characteristics of a long flight time, a fast speed, a large load, a stable structure, high reliability, etc., as shown in Figure 7. For the CW-25E UAV, the installation parameter is $\left[\begin{array}{c} \Delta x\\ \Delta y\\ \Delta z \end{array}\right]=\left[\begin{array}{c} 0.3121\;m\\ 0.00228\;m\\ 0.16917\;m \end{array}\right]$, i.e., the translation distance between the on-board camera and the center of the UAV.

**Figure 8:**
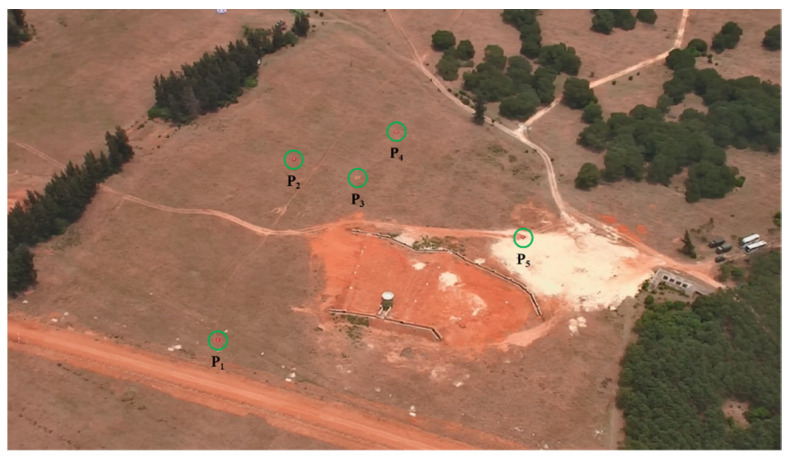
Image of target point.

The UAV flight altitude is 400 m, and 120 aerial photographs of the target area are taken on the set flight trajectory, and five 2 m × 2 m targets, P_1_~P_5_, are set as measurement points in the target area, as shown in Figure 8. The geographic coordinates of the measurement points are accurately measured using a GPS surveyor, whose time-differential positioning measurement accuracy is 0.1 m; thus, the measurement results can be used as the true value of the target’s geographic location. The positioning results are shown in Table 4.

**Figure 9:**
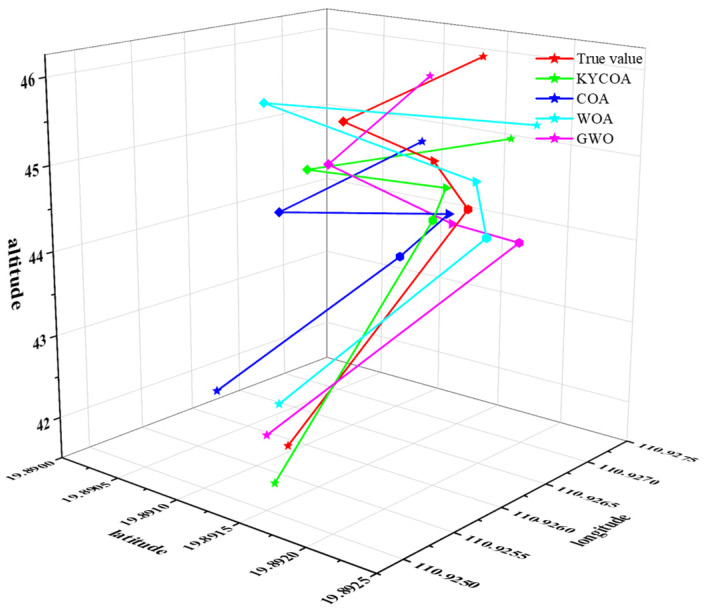
Schematic diagram of target point coordinates.

In order to analyze the enhancement of target localization accuracy with the KYCOA in real experiments, the experimental data were processed with the KYCOA, COA, WOA, and GWO; the coordinates of each point were averaged and compared with the true value, and the results of the experiments are shown in Figure 9, and the different coordinates of the points are labeled by different shapes.

**Figure 10:**
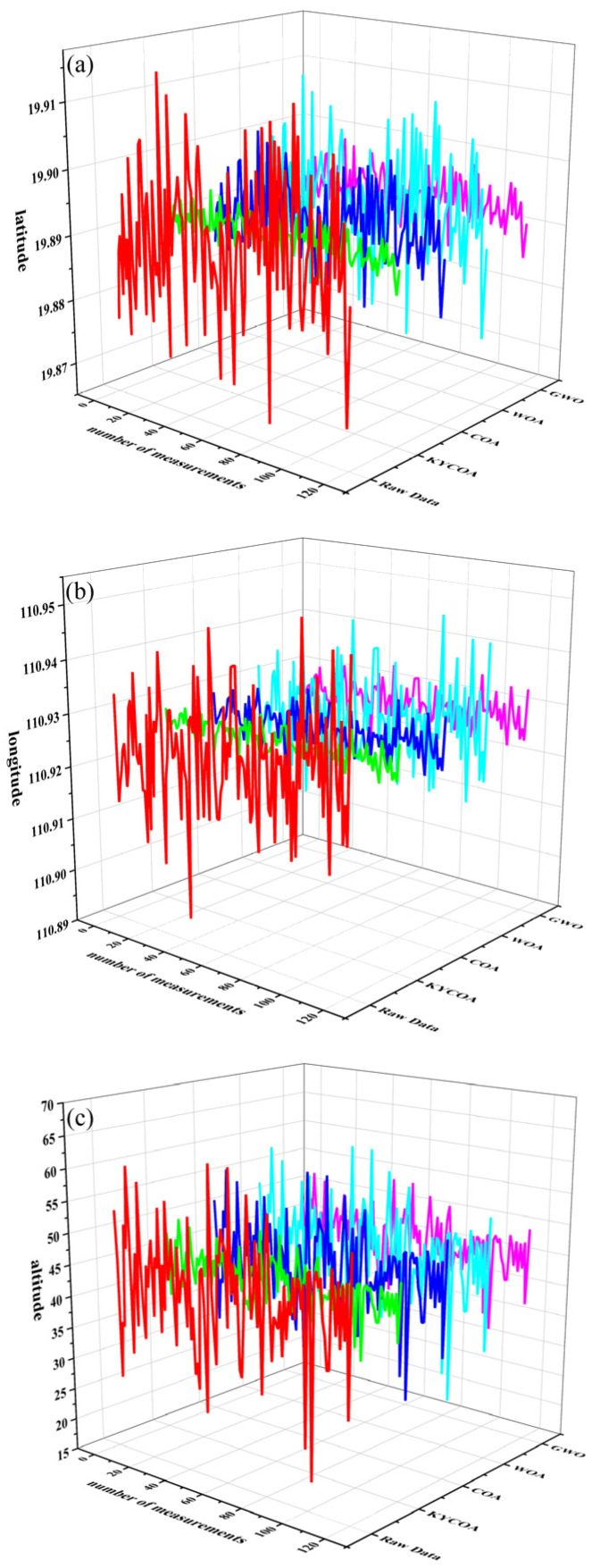
Comparison of latitude, longitude, and altitude distance errors for target point 1. (**a**) Latitude comparison chart. (**b**) Longitude comparison chart. (**c**) Altitude comparison chart.

Figure 10 shows the results of data processing performed 120 times for the UAV observation of target point 1, from which it is evident that the target point processed by the KYCOA is closer to the real value, where red represents raw data, green represents KYCOA-processed data, blue represents COA-processed data, cyan represents WOA-processed data, pink represents GWO-processed data.

Table 5 provides the error distance between the five target points and the true value point for each of the five target points.

From the data in Table 4, it is inferred that the KYCOA exhibits a significant error decrease compared to other data, which proves that the introduction of the KYCOA can reduce the error of target localization and improve the localization accuracy.

## 7. Conclusions

By constructing a coordinate system transformation model and obtaining the latitude and longitude information of the target area based on the camera pitch angle and azimuth angle as well as the UAV position and attitude angle in the image information, the nonlinear transmission law of the error of the UAV position, attitude angle, and camera parameters is obtained; furthermore, the synthetic error expression under the geocentric coordinate system is deduced. For the optimization of error allocation, the KYCOA that incorporates the good point set initialization and golden sine mechanism is proposed, which does not exhibit the defects of the traditional algorithm, i.e., low convergence accuracy and premature convergence, when dealing with the corresponding problems. Simulation results show that the KYCOA converges quickly within 200 iterations; its localization accuracy is significantly improved compared to that of the original algorithm; and its root mean square error is reduced by more than 50% compared to that of PSO and GWO. The practicality of the KYCOA is further verified by real flight tests, and the localization errors of the five target points are significantly improved compared to those of other algorithms. Future research will focus on the dynamic error allocation and optimization of algorithm hardware deployment in multi-UAV cooperative positioning scenarios to expand its application potential in complex tasks.

## Figures and Tables

**Table 1:** Simulation experiment data.

Parameters	Symbol	Standard Deviation	Value
UAV Latitude	B/°	0.0001	30°
UAV Longitude	L/°	0.0001	100°
UAV Height	H/m	5	400 m
UAV Yaw Angle	φ/°	0.2	\
UAV Pitch Angle	θ/°	0.2	0
UAV Roll Angle	σ/°	0.2	20°
Pod Heading Angle	α/mrad	2	−90°
Pod Pitch Angle	β/mrad	2	−25°

**Table 2:** Comparison of algorithm localization results.

	Truth Value	Raw Data	KYCOA	COA	WOA	GWO
Latitude\°	20	19.999633	19.999881	19.999791	19.999679	19.999832
Longitude\°	110	110.000011	109.999971	109.999977	109.999976	110.000018
Altitude\m	40	40.275	39.995	39.993	39.988	39.990
Distance\m	0	40.825	13.575	23.364	35.782	18.775

**Table 3:** Root mean square error results.

	Raw Data	KYCOA	COA	WOA	GWO
Latitude\°	0.24703	0.064617	0.10842	0.17047	0.082745
Longitude\°	0.24253	0.064714	0.11059	0.17178	0.085314
Altitude\°	126.19	52.16	78.24	130.4	104.32

**Table 4:** Real geographic location of the target.

Target Point	Latitude (°)	Longitude (°)	Altitude (°)
target point 1	19.89147036	110.9267209	44.8845
target point 2	19.89178402	110.9266519	44.4267
target point 3	19.89154581	110.9271469	46.0313
target point 4	19.89066431	110.9268279	45.1168
target point 5	19.89125446	110.9255049	41.8700

**Table 5:** Error distance.

Target Point	KYCOA (m)	COA (m)	WOA (m)	GWO (m)
target point 1	22.14	39.34	50.66	33.41
target point 2	26.50	45.73	55.09	35.86
target point 3	20.60	43.63	54.85	37.20
target point 4	27.17	47.48	57.46	40.22
target point 5	26.05	46.51	53.84	31.60

## Data Availability

The original contributions presented in the study are included in the article, further inquiries can be directed to the corresponding author.
